# Assessment of age changes and repeatability for computer-based rod dark adaptation

**DOI:** 10.1007/s00417-013-2324-5

**Published:** 2013-04-05

**Authors:** Laura Patryas, Neil R. A. Parry, David Carden, Daniel H. Baker, Jeremiah M. F. Kelly, Tariq Aslam, Ian J. Murray

**Affiliations:** 1 The Vision Centre, Carys Bannister Building, Faculty of Life Sciences, University of Manchester, Manchester, M13 9PL UK; 2 Vision Science Centre, Manchester Royal Eye Hospital, Manchester, UK; 3 University of Manchester Academic Health Science Centre, Manchester, UK; 4 Department of Psychology, University of York, York, UK

**Keywords:** CRT, Dark adaptometry, Rods, Ageing, Repeatability

## Abstract

**Purpose:**

To characterize the rate of rod-mediated sensitivity decline with age using a PC-driven cathode ray tube (CRT) monitor. To provide data regarding the repeatability of the technique.

**Methods:**

Dark adaptation was monitored for 30 min following a minimum 30 % pigment bleach, using a white 1° stimulus (modulated at 1 Hz), presented 11° below fixation on a CRT monitor. Thirty-three subjects with no ocular pathology and normal fundus photographs were divided into two groups: older (≥45, *n* = 16) and younger (<45, *n* = 17).

**Results:**

Rod recovery was assessed using component S2 of dark adaptation. S2 was significantly slower in the older (0.19 ± 0.03 log cd.m^−2^.min^−1^) compared with the younger group (0.23 ± 0.03 log cd.m^−2^.min^−1^, *t* = −4.05, *p* < 0.0003), despite no difference in visual acuity and fundus appearance. Faster rates of S2 recovery were correlated with lower threshold at 30 min (T_30_) (*r* = −0.49). Correlation coefficients between first and second measurements for S2 and T_30_ were 0.49 (*p* < 0.009) and 0.84 (*p* < 0.0001) respectively. The coefficient of repeatability was 0.07 log cd.m^−2^.min^−1^ for S2 and 0.35 log cd.m^−2^ for T_30_. The coefficients of variation for S2 and T_30_ were 15 % and 10 % respectively.

**Conclusions:**

Dark adaptation is slowed in normal ageing. CRT-based dark adaptometry is easily implemented and highly repeatable. The technique described in this article would be useful for documenting visual changes in future clinical trials assessing retinal health in the older eye with and without ocular pathology.

## Introduction

Dark adaptometry is considered a useful tool for investigating a variety of systemic and ocular diseases including vitamin A deficiency [1], liver disease [2], diabetes [3, 4], age-related macular degeneration (AMD) [5–10], retinitis pigmentosa [11] and congenital stationary night blindness [12]. It has also been used to assess non-pathological mechanisms of ageing [13, 14]. The term 'dark adaptation' refers to the gradual recovery of visual sensitivity in total darkness following exposure to a bright light. The light bleaches the photoreceptor visual pigment, resulting in its inactivation and a profound (∼5 log units) loss of sensitivity. Classically, the dark adaptation function has been described as biphasic, and comprises an initial rapid phase subserved by the cones, followed by a slower phase subserved by the rods. In recent years, significant advances have been made in our understanding of the biological processes underpinning rod recovery [15]. In terms of analysing and modelling dark adaptation data to obtain clinically useful parameters, the rod recovery can be partitioned into three partly overlapping components: S1, S2, and S3 [16]. Normally, S1 is obscured by cone recovery so that, in the standard dark adaptation curve, S2 is the first measurable sign of rod recovery.

Slowed dark adaptation, particularly the rate of S2, is characteristic of ageing and AMD, and precedes retinal changes and cone-mediated visual function changes such as reduced visual acuity (VA) [8, 9, 14, 17, 18]. Dark adaptometry is, therefore, likely to become the test of choice for investigating ageing and assessing efficacy of therapies and management strategies for early stage AMD. To that end, an inexpensive, readily available and repeatable technique for measuring dark adaptation kinetics will be essential if functional, as well as structural, features form part of the clinical outcomes.

There have been many studies aimed at using dark adaptation to assess age-related ocular pathology, but few have provided data regarding the repeatability of the slope of S2. Accurate determination of this parameter’s repeatability is important, because detecting small changes in the slope of S2 is of clinical significance. The problem of accurate determination of dark adaptation parameters is compounded by the fact that data obtained from elderly subjects, who may or may not have ocular pathology, are usually more variable than those produced by young, healthy individuals. Repeatability and reliability of any technique will, therefore, be paramount to its applicability.

Two recent studies employed cathode ray tube (CRT) technology to assess dark adaptation kinetics [18, 19]. CRTs are ideally suited to, and used extensively in, visual psychophysics research. Their temporal and spatial characteristics are well-documented, and they are easily controlled by a computer. A major limitation of using computer monitors for dark adaptation, however, is that they have a limited dynamic range, but this problem can be avoided by the use of neutral density (ND) filters [18].

As far as we are aware, the coefficient of repeatability (CoR) for the rod parameters measured by CRT dark adaptometry has not yet been established. The CoR is important when evaluating the performance of an instrument that is used to detect clinically significant changes over the course of an intervention trial [20]. In this study, we use a customized version of commercially available software to investigate the ability of CRT-based dark adaptometry to quantify delays in rod-mediated recovery in ageing. We also provide data regarding the repeatability of this technique.

We chose to focus on the measurement of the slope of S2, since rods are more vulnerable than cones in ageing and AMD [8, 9, 21].

## Methods

### Subjects

Thirty-three normal volunteers participated in this study, and were divided into two groups. The older group (≥45 years old, age range 45–68, mean 57.44 ± 7.98, *n* = 16) consisted of eight males and eight females. The younger group (<45 years old, age range 15–36, mean 25.12 ± 6.08 *n* = 17) consisted of ten males and seven females.

Younger subjects were primarily recruited from the University of Manchester undergraduate population and older subjects from university staff. Informed consent was obtained. The tenets of the Declaration of Helsinki were followed. This study was approved by the University of Manchester Committee on the Ethics of Research on Human Beings.

All subjects had recently had an eye examination (up to 12 months before recruitment), were free from any ocular disease (e.g., glaucoma, AMD, cataract) and were not taking nutritional supplements. Subjects with diabetes or liver disease, current smokers, and those using systemic medications known to be retinotoxic were excluded from the study.

On the day of testing, all subjects underwent assessment of VA and dark adaptation. Fundus photographs were taken with a TRC-NW6S Non-Mydriatic Retinal Camera (Topcon, Tokyo, Japan). The VA was measured using an internally illuminated Early Treatment of Diabetic Retinopathy Study (ETDRS) chart (166.3 ± 3.92 cd.m^−2^), and expressed as logarithm of the minimum angle of resolution (logMAR).

The fundus photographs were processed using IMAGE Net 2000 software (Topcon, Tokyo, Japan) and viewed on a 20-in. monitor (1,600 × 1,200 pixels, 32 bits). The fundus images of all participants were graded by one of the authors (LP) according to a macular grading scale [14]. No subject had a grade beyond 1, thus all were classified as being normal.

### Procedure

**Fig. 1:**
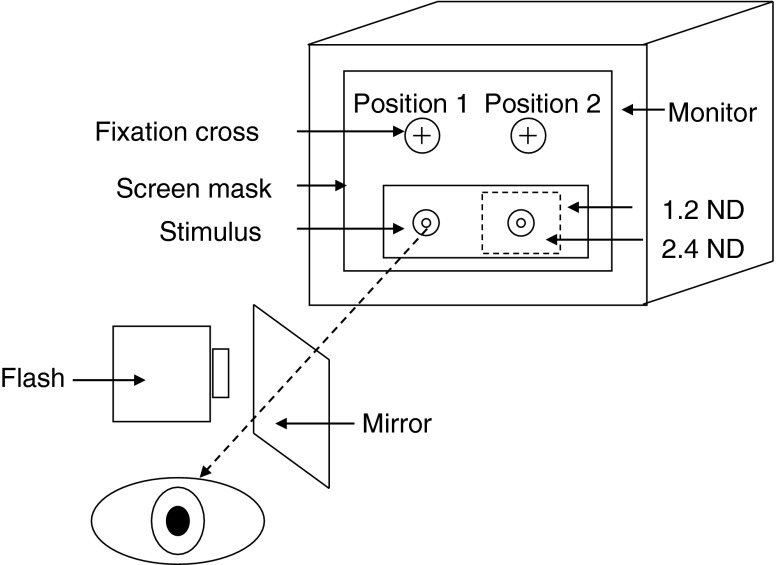
The experimental set up. A mask with four apertures corresponding to the stimuli and fixation cross locations covered the entire screen. A 1.2 log unit ND filter was attached to the back of the mask at stimulus positions 1 and 2. When the filtered screen luminance fell below −2.3 log cd.m^−2^, the fixation cross and the stimulus were extinguished at position 1 and moved to position 2, where an additional smaller 2.4 log unit ND filter (attached to the back of the mask) exposed the remaining region of rod recovery. The retinal area to be tested was accurately bleached by aligning the flash with the stimulus (at position 1) through the use of a semi-silvered mirror

The stimuli were generated using a visual stimulus generator (VSG 2/5, Cambridge Research Systems, Rochester, UK) running Visual Psychophysics Engine software (Cambridge Research Systems, customized by NRAP) and presented on a calibrated and gamma-corrected high-resolution CRT monitor (Sony GDM-F500R, Tokyo, Japan). A black cardboard mask with four apertures corresponding to the stimuli and fixation cross was placed over the monitor screen. One or more 1.2 log unit ND filters (#299; Lee Filters, Andover, UK) were placed in front of the test stimulus in the configuration illustrated in Fig. 1.

The observer fixated a red cross (0.3°) at position 1, and responded to a 1° circular test spot (1931 CIE *x* = 0.31, *y* = 0.316), temporally modulated with a 1 Hz square wave and presented at 11° in the inferior field (a location typically used in the standard Goldmann–Weekers adaptometer). The stimulus intensity was attenuated by a 1.2 log unit ND filter that extended over both stimulus locations. When the absolute filtered intensity of the stimulus was below −2.3 log cd.m^−2^ (usually after about 12–15 min), the fixation cross and stimulus at position 1 were extinguished and re-appeared in position 2. At this location, the stimulus was further attenuated by a 2.4 log unit ND filter, so that total attenuation for the latter stages of the procedure was 3.6 log units. The dynamic range was sufficient (approximately 5.5 log units) to enable the measurement of the entire scotopic recovery function. A similar approach has been used previously with the filters mounted on goggles worn by the observer [18, 19]. In our procedure, the expansion of the dynamic range by addition of further ND filters is fully automatic. In the absence of other visual cues (as the subject is in total darkness), the shift in location of the targets is rarely noticed.

All subjects were dark-adapted for 5 min, followed by a practice session for a further 5 min. A localised 30–98 % visual pigment bleach [22] was then performed using an electronic 0.9 ms flash of white light (Nikon Speedlight SB-800, Tokyo, Japan). The flash intensity was 6.08 log cd.s.m^−2^, as measured using a PR1500 spot photometer (Photo Research, Burbank, CA, USA). Since the rate of S2 is independent of the bleach magnitude provided the bleach is greater than 10–20 % [15], we performed the measurements on natural pupils.

The flashgun was positioned 15 cm from the eye, and at this distance subtended an angle of 20.9° wide by 13.3° high. The flash and the bleach area were precisely aligned so that location of the test stimulus was centred on the bleached area of the retina. This was achieved by using a calibrated semi-silvered mirror, as illustrated in Fig. 1, so that the subject observed the fixation mark when the flash was fired. An adjustment of 0.3 log units was made to all thresholds to compensate for the absorption characteristics of the mirror, which remained in place throughout the experiment.

Monocular thresholds were measured in complete darkness in a purpose-built room immediately after bleaching. The experiment was controlled by a computer external to the dark room. Stimulus luminance was reduced in steps of 0.1 log unit until the subject reported its absence. Thresholds were set approximately twice per minute for a duration of 30 min. The non-stimulated eye was patched during testing, and the subjects wore their best optical correction for the test distance. The subject’s head was positioned in a chin/head rest. All participants repeated the dark adaptation measurement twice, separated by at least 1 week. The data presented hereafter are the means of two visits.

### Data analysis

Dark adaptation curves were plotted as log_10_ threshold in cd.m^−2^ versus time in minutes. These were fitted with a single exponential component to the cone phase and two linear components to the rod phase, as described by McGwin et al. [23]. The non-linear regression technique was implemented in Matlab (Mathworks, MA, USA) and yielded the following parameters of the dark adaptation curve: cone recovery rate, cone threshold, the rod–cone break (RCB), the slopes of the second (S2) and third (S3) rod components, the transition point between the two, and the threshold 30 min after the bleach (T_30_). Of these parameters, we were primarily interested in component S2 and T_30_. The latter was corrected for pre-retinal absorption (pupil diameter and media opacity) based on previous work [24–27].

Kolmogorov–Smirnov tests were used to determine that the distributions of all dark adaptation parameters did not differ from normal. Origin^@^ (Northampton, MA, USA) and Matlab were used for statistical analysis and graph plotting. Repeatability was assessed using the standard correlation coefficient (Pearson’s *r*) and by calculating the CoR (1.96 multiplied by the standard deviation of the differences between test and retest data) and coefficients of variation (CoV, the ratio of the standard deviation to the mean multiplied by 100). Independent sample *t*-tests were used to make comparisons between groups (younger vs older, males vs females).

## Results

### Preliminary data

**Fig. 2:**
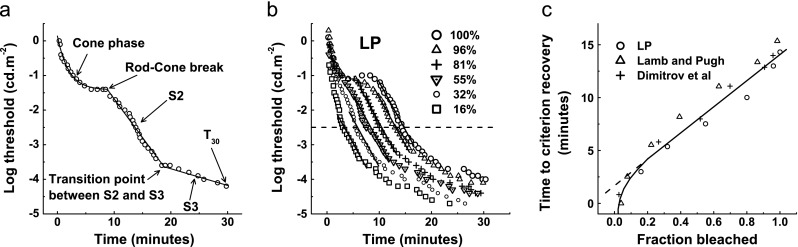
**a** Typical dark adaptation data measured with our CRT-based technique for a young, healthy observer (LP) measured inferiorly at 11° degrees eccentricity, using a 1° white light stimulus following an 82 % bleach. The data points were fitted with an exponential-bilinear model. *S2* is the second rod component, *S3* is the third rod component and *T*_*30*_ is the threshold 30 min after the bleach. SSE = 0.3, *r* ^2^ = 0.9. **b** Dark adaptation curves for the same observer using the same technique following a range of bleaches (16–100 %). The *parallel solid lines* plot component S2 and demonstrate a constant rate of rod recovery across bleaches. The *horizontal dashed line* is an arbitrary criterion (−2.5 log units) used to plot the graph in panel **c**. **c** Linear relationship between fraction bleached (above 20 %) and the time required to reach a criterion recovery level for our data (LP) and those from previous studies

Figure 2a depicts a classic dark adaptation function obtained with our CRT-based technique for a young, healthy observer (LP, one of the authors). An exponential-bilinear model partitioned the curve into three distinct phases of sensitivity recovery: a cone-mediated phase, followed by a rod-mediated phase divided into two linear regions. The two components of rod dark adaptation, S2 and S3, had negative slopes of 0.24 log cd.m^−2^.min^−1^ and 0.06 log cd.m^−2^.min^−1^ respectively.

The parallel lines in Fig. 2b plot component S2 for different bleach intensities. There was no significant correlation between bleach and slope of S2 (*r* = 0.65, *p* > 0.23), confirming that this phase of rod recovery is independent of the bleach magnitude provided that the bleach is greater than 10–20 %. In Fig. 2c, the time taken to reach an arbitrary threshold of −2.5 log units (extracted from Fig. 2b) was re-plotted against the fraction bleached. The straight line fit when plotted in semi-logarithmic co-ordinates for bleaches greater than 10–20 % reveals the rate-limited behaviour of S2.

### Repeatability

**Fig. 3:**
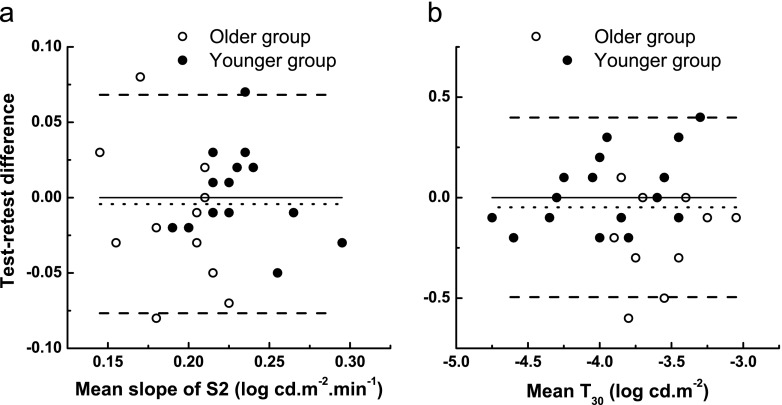
Test–retest differences versus means to assess the repeatability of dark adaptation curve parameters *S2* (panel **a**) and *T*_*30*_ (panel **b**). The *dotted line* represents the bias (test-retest mean differences) and the *dashed lines* represent 95 % limits of agreement

In order to quantify measurement error, repeated measurements were obtained on different days. The final three columns of Table 1 summarize correlation coefficients, CoRs, and COVs for the following parameters: RCB, S2, S3, and T_30_. Of these, we were primarily interested in S2 and T_30_. Correlation coefficients between first and second measurements for S2 and T_30_ were 0.49 (*p* < 0.009) and 0.84 (*p* < 0.0001) respectively. The average absolute change between sessions (dotted line in Fig. 3) was 0.004 (±0.04) log cd.m^−2^.min^−1^ for S2 and 0.05 (±0.23) log cd.m^−2^ for T_30_, indicating only minimal bias. The CoR was 0.07 log cd.m^−2^.min^−1^ for S2 and 0.35 log cd.m^−2^ for T_30_. The CoV was 15 % for S2 and 10 % for T_30_.

**Table 1:** Summary of statistical comparisons: older vs younger group and test–retest repeatability

Parameter	Older group mean (±SD)	Younger group mean (±SD)	*P* value^a^	Test–retest correlation coefficient, (*p* value)	Test–retest CoR	Test–retest CoV
RCB (mins)	7.20 (2.45)	7.04 (1.53)	0.82	0.67 (<0.0001)	3.62	28 %
S2 (log cd.m^−2^.min^−1^)	0.19 (0.03)	0.23 (0.03)	<0.0003	0.49 (<0.009)	0.07	15 %
S3 (log cd.m^−2^.min^−1^)	0.04 (0.02)	0.05 (0.02)	0.05	0.34 (0.05)	0.05	40 %
T_30_ (log cd.m^−2^)	−4.34 (0.25)	−4.40 (0.44)	0.63	0.84 (<0.0001)	0.35	10 %

^a^Independent *t* test older vs. younger group

### Dark adaptation in older and younger eyes

**Fig. 4:**
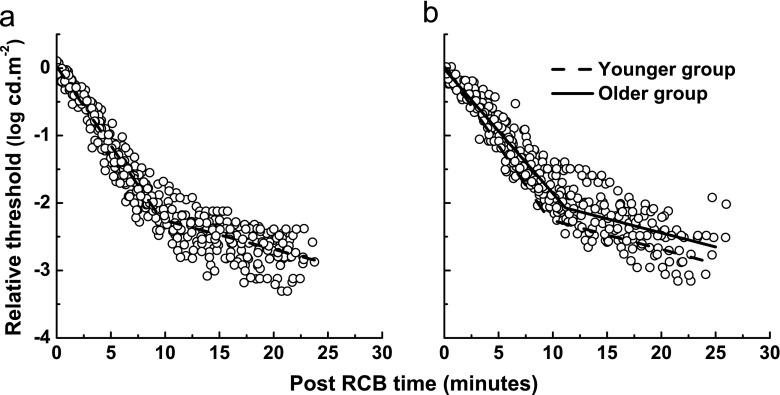
Group data showing the S2 and S3 regions of rod recovery for younger (**a**) and older (**b**) subjects. A bilinear function was fitted to each data set. The younger group model (*dashed line*) is superimposed onto the older group data in **b** to demonstrate slowing of the S2 region and elevated thresholds in the older group. Data have been shifted along the *x* and *y* axes so that the individual RCBs were coincident

VA in the test eye for all subjects was at least 0.2 logMAR, and there was no difference in VA between the two groups (*t* = 1.00, *p* = 0.3). Figure 4 depicts rod dark adaptation kinetics (components S2 and S3), after the RCB, for the younger and the older group. Each subject’s curve was linearly shifted in *x* and *y* directions, so that their individual RCBs were coincident. The group data were fitted with a bilinear function. The older group (solid line, Fig. 4b) had a shallower slope of S2 compared with the younger group (dashed line), indicating slower rate of recovery. The vertical (upward) shift in the older group along the *y*-axis indicates threshold elevation across the entire rod-dominated region of sensitivity recovery.

**Fig. 5:**
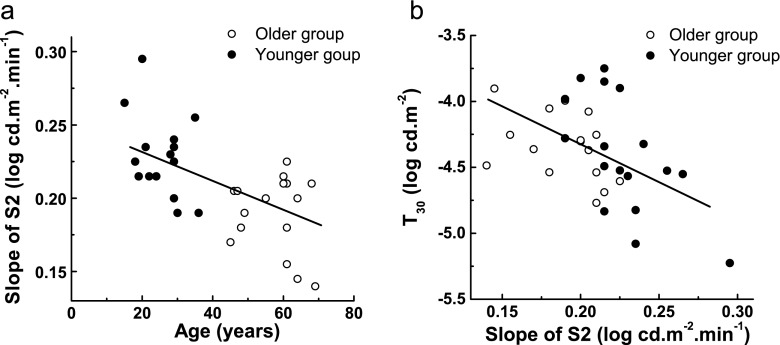
**a** Scatter plot of S2 as a function of age. The *line* represents linear regression fitted to the data (*r* = −0.62, *p* < 0.0002). **b** Scatter plot with a line of best fit illustrating negative correlation between *T*_*30*_ and *S2* (*r* = −0.49). All data points are means of two sessions. Thresholds were corrected for lens density and pupil miosis

A summary of statistical comparisons between the two groups for RCB, S2, S3, and T_30_ is presented in Table 1. The younger group had an average S2 of 0.23 ± 0.03 log cd.m^−2^.min^−1^, with a time constant (*τ* = log_10_ (e)/S2) of 1.9 min. The older group was significantly slower than the younger group (*t* = −4.05, *p* < 0.0003), with an average S2 of 0.19 ± 0.03 log cd.m^−2^.min^−1^ (*τ* = 2.3 min). The negative correlation between S2 and age (*r* = −0.62, *p* < 0.0002) is shown in Fig. 5a. The rate of recovery over the S2 region decreased 0.01 log units/min per decade. S2 was also correlated with T_30_ after corrections for media changes (*r* = −0.49), as illustrated in Fig. 5b.

Before pre-retinal correction, T_30_ was elevated in the older group by 0.4 log units (*t* = 3.14, *p* < 0.004). However, after pre-retinal correction, the older group sustained a non-significant threshold elevation of 0.1 log units compared with the younger group (*t* = −0.48, *p* = 0.63). We did not observe any significant gender differences for S2 (*t* = 0.28, *p* = 0.79) and T_30_ (*t* = 0.29, *p* = 0.77) in our cohort. As shown in Table 1, there was no significant difference between the older and younger group for RCB (*t* = 0.23, *p* = 0.82) and S3 (*t* = −2.03, *p* = 0.05).

## Discussion

The data presented in this paper demonstrate that our CRT-based dark adaptometry produces results that agree with previous studies [15, 18]. The slowing of component S2 with increasing age found in this study was 0.01 log units/decade, and reflects reduced rhodopsin regeneration rate, in agreement with other psychophysical [14] and rod densitometry [28] data. The technique proved capable of differentiating between younger and older eyes (for the S2 parameter), despite no differences in VA and fundus appearance between the two groups. The power in this study to detect a difference between old and young eyes was 0.96 (calculated using G*Power 3.0.10). This is a good indicator of the ability of the technique to detect small changes in the slope of S2, either between two groups or in individuals in a longitudinal study.

Prolonged dark adaptation kinetics in older adults lead to difficulties with vision-oriented tasks in dim lighting, and increase the risk of night-time falls and road traffic accidents. These problems have been confirmed in self-reporting surveys such as that described by Scilley et al. [29], who used a questionnaire designed specifically for assessing low-light visual problems. Difficulties arise, however, in establishing the exact contribution of impaired night vision to accidents, because of the absence of a satisfactory test which can be used routinely under clinical conditions. It seems likely that those older observers with a healthy lifestyle and good nutrition can be expected to have relatively good scotopic recovery, but confirming such a hypothesis might be difficult, because a technique for measuring the slope of S2 precisely is not generally available. The method described here could be used to quantitatively assess patient night vision with excellent reproducibility, enabling researchers to use scotopic recovery as a realistic outcome measure.

The correlation we found between the rate of rod-mediated recovery (slope of S2) and T_30_ is at odds with one previous study [30]. In that previous study, the absolute threshold was measured which may not be directly comparable to our measure of threshold after 30 min. Our correlation can be explained by geometry of the dark adaptation function and by the cellular model of recovery kinetics presented by Lamb and Pugh [15]. If the slope of S2 is steeper, then the threshold at 30 min will be lower. Although the measurements were restricted to 30 min, and some observers would have reached lower thresholds had the time been extended, it seems likely that the rate of S2 and T_30_ share the same cellular and molecular mechanisms [15].

The CRT method has previously been compared with the conventional Goldmann–Weekers adaptometer (GWA), showing good agreement between the two methods on almost all parameters of the dark adaptation curve including cone recovery rate, RCB and S2 [18]. The general problem with the GWA is its poor repeatability for cone recovery. Gaffney et al. [31] have shown a clinically unacceptable CoR for cone recovery time constant, and concluded that the GWA would not be a useful instrument for documenting visual changes in future clinical trials. Christoforidis and Zhang [32] also used GWA in a test–retest paradigm. They showed no learning effects and no statistically significant differences on repeated measures for any of the parameters of the scotopic recovery curve. Although their group mean S2 recovery rate of 0.15 log cd.m^−2^.min^−1^ is slower than ours and that typically reported in the literature for healthy subjects, their CoR for S2 of 0.06 log cd.m^−2^.min^−1^ is very similar to ours.

Dimitrov et al. [18] used a similar method to the one described in this article. However, in that study, which also investigated AMD patients, the CoV for the rod parameters was not given. Their CoV for the RCB was 32 % for normal and 44 % for AMD subjects, which is slightly higher than our COV of 28 %. Of note is the considerably larger COV for RCB and S3 than for S2 and T_30_ in the present study. This could be due to the fact that, unlike S2, the RCB and S3 are largely dependent on the magnitude of the bleach [15], which highlights the importance of precise and uniform bleaching between visits for longitudinal clinical trials. In the present study, we were able to accurately bleach the area to be tested by using a semi-silvered mirror. Although we did not dilate the pupils because we were primarily interested in the slope of S2 (and used bleaches ≥30 %), we would highly recommend pupil dilation in future clinical trials investigating multiple dark adaptation parameters. This is because dilation of pupils allows tighter control over the bleach.

Finally, our technique readily elicited the third rod component (S3) unlike the protocol suggested by Dimitrov et al. [18] using a single 2.6 log unit ND filter. Such a narrow range may pose problems in evaluating dark adaptation in ageing, particularly in subjects with good scotopic sensitivity due to its ceiling effect.

In summary, the results of this study demonstrate the validity of using an easily implemented computer-based technique to explore scotopic sensitivity recovery in ageing, using an automated and inexpensive method of expanding the luminance range with ND filters. The method is highly repeatable for the measurement of rod-mediated dark adaptation parameters (S2 and T_30_), without requiring pupil dilation. Because of its sound physiological basis, S2 is of particular interest. It seems likely that given its many advantages, dark adaptometry based on digital methods will become the method of choice for future work in assessing retinal health in the older eye with and without ocular pathology.
